# Comparison of 180° and 360° Arc Data Acquisition to Measure Scintigraphic Parameters from Gated Single Photon Emission Computed Tomography Myocardial Perfusion Imaging: Is There Any Difference?

**DOI:** 10.4274/mirt.96720

**Published:** 2016-02-10

**Authors:** Hamid Javadi, Ali Mahmoud-Pashazadeh, Mehdi Mogharrabi, Darioush Iranpour, Abdollatif Amini, Mohammadreza Pourbehi, Mehdi Akbarzadeh, Iraj Nabipour, Majid Assadi

**Affiliations:** 1 Golestan University of Medical Sciences, Golestan Research Center of Gastroenterology and Hepatology, Gorgan, Iran; 2 Bushehr University of Medical Sciences, The Persian Gulf Nuclear Medicine Research Center, Bushehr, Iran; 3 Bushehr University of Medical Sciences, Bushehr Medical Center Hospital, Department of Cardiology, Bushehr, Iran

**Keywords:** 180° data collection, 360° data collection, ejection fraction, end diastolic volume, end systolic volume, gated myocardial perfusion

## Abstract

**Objective:**

The aim of the current study was to compare 180° and 360° data collection modes to measure end diastolic volume (EDV), end systolic volume (ESV) and ejection fraction (EF) values of the cardiac system by gated myocardial perfusion tomography.

**Methods:**

Thirty-three patients underwent gated myocardial perfusion tomography. Single photon emission computed tomography data of patients’ heart were acquired by 180°, 45° left posterior oblique to 45° right anterior oblique, and 360° to obtain EDV, ESV, EF and cardiac volume changes (V1, V2, V3, V4, V5, V6, V7 and V8) throughout each cardiac cycle.

**Results:**

Results of the current study indicated that there were no significant differences between 180° and 360° angular sampling in terms of measuring EDV, ESV and EF in myocardial perfusion imaging. Cardiac volume change patterns during a cardiac cycle were also similar in 360° and 180° scans. We also observed that there was no difference in EDV, ESV and EF values between the group with stress induced by exercise and the group with stress imposed by dipyridamole.

**Conclusion:**

As there is no difference between 180°and 360° cardiac scanning in terms of EDV, ESV and EF, half-orbit scan is recommended to study these cardiac system parameters because it offers more comfort to patients and a shorter scanning time.

## Abstract (tr)

**Amaç:**

Bu çalışmanın amacı gated miyokard perfüzyon tomografisinde kardiyak sisteme ait end-diastolik volüm (EDV), end-sistolik volüm (ESV) ve ejeksiyon fraksiyonu (EF) ölçümlerinde 180° ve 360° veri toplama yöntemlerini karşılaştırmaktır.

**Yöntem:**

Otuz dokuz hastaya gated miyokard perfüzyon tomografisi uygulandı. Hastaların kardiyak tek foton emisyon bilgisayarlı tomografi verileri 180°, 45° sol posterior oblikten 45° sağ anterior oblik’e, ve 360° ile elde edilerek EDV, ESV, EF ve her kardiyak siklüs boyunca oluşan kardiyak volüm değişiklikleri (V1, V2, V3, V4, V5, V6, V7 ve V8) saptandı.

**Bulgular:**

Bu çalışmanın sonuçları miyokard perfüzyon görüntülemesi ile EDV, ESV ve EF ölçümleri için 180° ve 360° açısal örnekleme arasında anlamlı fark olmadığını göstermiştir. Kardiyak siklüs boyunca oluşan kardiyak hacim değişiklikleri de 360° ve 180° görüntülemede benzer bulunmuştur. Egzersizle veya dipiridamol ile stress oluşturulmuş gruplar arasında da EDV, ESV ve EF değerleri açısından farklılık olmadığı tespit edilmiştir.

**Sonuç:**

180° ve 360° kardiyak görüntülemede EDV, ESV ve EF değerleri açısından farklılık olmadığından, kardiyak parametreleri değerlendirmek için hastalar açısından daha konforlu olan ve daha kısa sürede tamamlanan yarı-eksenli görüntülüme önerilmektedir.

## INTRODUCTION

In the late 1980s when electrocardiography (ECG) has been successfully applied in conjunction with single photon emission computed tomography (SPECT) scanner, gated myocardial perfusion SPECT or gated SPECT has been introduced as a valuable technique to study cardiac function. Study of the functional abnormalities of the myocardium by gated SPECT can be used to assess the level of associated risks and adopt appropriate therapeutic strategies (1). In this technique, cardiac performance is studied after injection of a radiotracer and its take-up by the myocardium based on changes in both the geometry and the rate during a cardiac cycle, while an ECG system is used and mapped to the scan data (2,3). Gated myocardial perfusion SPECT provides valuable information for physicians who are dealing with patients with coronary artery disease (CAD) to better diagnose myocardium pathologies (4), to assess related risks (5,6,7), to evaluate cardiac viability (8), and to follow-up patients after revascularization procedure (9,10). In the last years, this method has been widely used in the clinical setting for a variety of reasons. First, in gated SPECT it is possible to simultaneously study both the perfusion and the function of the left ventricle (11,12). The flexible protocol of this method, mainly due to the kinetic properties of sestamibi and tetrofosmin that are tracers labeled with 99mTc, is the other reason that has made it a suitable method to evaluate patients with CAD (13). Recent advances in SPECT technology and related computer systems have led to a shortened acquisition time and efficient processing methods, consequently to improved application of gated SPECT (14). In addition to all these advantages, results of the myocardium studies by gated SPECT have been validated against other modalities used for cardiac imaging (15,16,17).

The end diastolic volume (EDV), end systolic volume (ESV) and left ventricular ejection fraction (EF) data acquired in gated myocardial perfusion imaging are important parameters in determining the functional status of the myocardium (18,19).

Generally, two types of data sampling are used in gated SPECT; 180° acquisition and 360° acquisition. Because of the anatomical location of the heart, which is the left anterior side of the thorax, photons coming from the right posterior side is attenuated significantly, degrading the quality of image obtained by 360° tomographic acquisitions (18). The 180° arc acquisition from the left posterior to the right anterior, which is proposed to have advantages over the other method in detecting myocardial abnormality, is suggested as an alternative to 360° circular acquisition.

Therefore, in this study we aimed to quantitatively compare 180° with 360° angular SPECT by using EDV, ESV, EF and cardiac volume changes over an R-to-R interval in normal and ischemic subjects. Furthermore, the effects of stress either induced by exercise or by a pharmacologic agent on these parameters were evaluated in both imaging modes.

## MATERIALS AND METHODS

Thirty-nine patients (18 males and 21 females) with an average age of 51.77 (range of 33 to 75) years were included in this study. Prior to the study, all patients were informed about the procedure and informed consent forms were obtained. To perform SPECT study, all participants were injected with 740 MBq of ^99m^Tc-sestamibi. The study was conducted at the stress condition that was induced by either exercise or dipyridamole injection, to obtain EDV, ESV and EF values of the patients.

In stress imaging by exercise, which was performed on 23 individuals, patients underwent treadmill testing during which they received ^99m^Tc-sestamibi in the peak of the exercise. The stress imaging was performed about 15 to 45 minutes after injection. In the stress imaging by pharmacologic agent, which was performed on 16 cases, patients received infusion of 0.56 mg/kg of dipyridamole over 4 minutes. 99mTc-sestamibi was injected approximately three minutes after completion of this infusion.

Gated myocardial perfusion studies were acquired for 8 frames/cardiac cycle projections over 180° and 360° using a SPECT scanner (ADAC-Pegasys). Acquisition parameters were set as a beat acceptance window at 20% of the average R-R interval, a 140-keV photopeak with a 20% window and a matrix size of 64×64.

Paired Student’s t tests were applied to determine significant differences in the data. A p value less than 0.05 was defined as statistically significant.

## RESULTS

Based on the scan of 39 cases included in this study, 12 patients were detected to have ischemia and 27 cases were considered as normal. The EDV, ESV and EF values as measured by myocardial perfusion imaging in these two groups by 180° and by 360° data collection are presented in Table 1. Analysis of the EDV, ESV and EF showed that there was no significant difference in these parameters between the two modes of imaging (p>0.05).

In addition, EDV, ESV and EF values measured by both 180° scan and 360° scan in stress-exercise and stress-dipyridamole stages were compared and presented in Table 2. Analysis of the data revealed that there were no differences between these two methods to induce stress in patients with respect to EDV, ESV and EF (p>0.05). This finding was detected in both 180° scan and 360° scan.

For all patients, the R-to-R interval and relevant bins were also measured. The findings indicated that there was no significant difference between normal subjects (8.00±0.024 ms) and ischemic patients (8.00±0.022 ms) (p>0.05).

**Figure 1:**
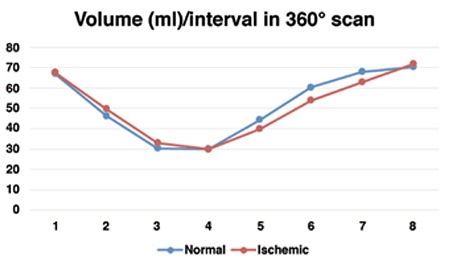
Volume changes over a cardiac cycle measured by 180° and 360° scan modes

Changes of cardiac volume over a cycle that was measured in eight frames, for both 180° and 360° scan modes, are presented in Figure 1. It is evident that the change patterns were the same. While the curve related to 360° orbit in stress conditions were lower in all eight volumes as compared to 180° orbit, the difference between corresponding volumes were not significant (p>0.05).

**Figure 2:**
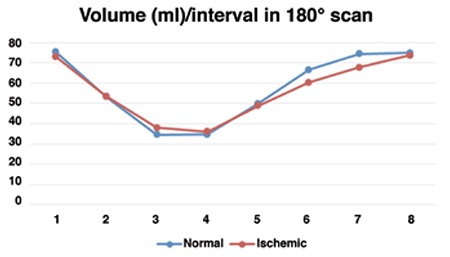
Volume changes over a cardiac cycle in normal and ischemic patients measured by 180° scan mode

**Figure 3:**
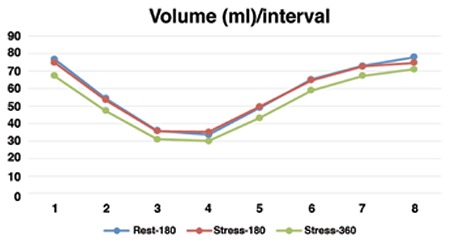
Volume changes over a cardiac cycle in normal and ischemic patients measured by 360° scan mode

Volume-time curves over a cardiac cycle for normal and ischemic patients was almost similar and there was no significant difference between the volumes of corresponding phases (p>0.05). In 180°data sampling, shown in Figure 2, volume curves of normal and ischemic patients showed similar decreasing trend to reach to the ESV phase. After that, in spite of the lower volumes observed for ischemia curve in V6 (60.42 vs 66.59) and V7 (67.83 vs 74.59), the differences were not significant. In 360°data sampling, presented in Figure 3, the pattern of normal and ischemic curves was almost the same in the decreasing part. After ESV, while lower cardiac volumes were observed in V5 (39.92 vs. 44.43), V6 (53.92 vs. 60.43) and V7 (62.83 vs. 68) in ischemic patients as compared to normal subjects, the differences were not statistically significant. Ischemia, when compared to normal myocardium, did not change cardiac volumetric function over a cycle.

## DISCUSSION

The main aim of the present study was to quantitatively compare 180° and 360° angular sampling SPECT for the assessment of EDV, ESV, EF and volume changes over a cardiac cycle. Participants of this study were divided into two groups of normal subjects and ischemic patients, then their EDV, ESV and EF values were assessed by 180° and 360° data collection methods. We observed that in myocardial image perfusion study, there was no significant difference between 180° and 360° orbits in terms of EDV, ESV and EF values. This finding indicates that measurement of the main parameters in myocardial perfusion imaging by using 180° data collection orbit may lead to the same results as obtained by 360° technique. In addition to the more favorable image quality that can be achieved in 180° scan, the required data can be obtained in a relatively shorter time in comparison with 360° orbit. Therefore, based on the results of the current attempt for a specific cardiac study, it can be concluded that 360° acquisition offers no significant advantages over 180° acquisition to evaluate myocardial function during gated myocardial perfusion. This finding is in concordance with the results of a recently published similar study (18).

Until now, various attempts have been done to compare the two scan modes of 180° and 360° in myocardium SPECT study from different aspects. Knesaurek et al. (20) investigated effects on geometric distortion in 180° and 360° data acquisition methods. They reported that there was more distortion of point source to ovals in 180° than in 360° data sampling. While geometric distortion was the same for both imaging modes on coronal and sagittal sections, it was less in 360° mode in comparison to 180° on transverse sections. In a study performed by Liu et al., (21) the effect of 180° and 360° acquisition orbits on the homogeneity of images obtained by 99mTc SPECT was evaluated on a phantom. They found that SPECT study of the myocardium using a 180° orbit could lead to inhomogeneity in the resulting image. In case of off-centered heart, which is usual in clinical practice, this effect could even be more highlighted and the defect size detected in Magnetic particle inspection could be well overestimated. Application of a full 360° circular data collection may have advantages over 180° imaging mode by avoiding related myocardial defects and producing information that is more accurate. In another study conducted by Coleman et al. (22) on both phantoms and patients, results of the 201Tl cardiac SPECT were compared between 180° mode without attenuation correction and 360° scan mode with attenuation correction. They concluded that the 360° scan mode, because of attenuation correction, was more suitable for 201Tl studies. In this mode of imaging, the variability related to poor counting statistics was also less expressed, and the image contrast of patients’ myocardium was similar to that obtained by 180° data collection. For single-headed camera systems, 360° data acquisition may offer time advantages when compared with 180° data sampling, although this is not the case for dual-head gamma cameras.

On the other hand, a study comparing 180° and 360° data collection in myocardial 99mTc-SPECT on 12 patients by Maublant et al. (23) indicated that 360° data acquisition technique suffers a main disadvantage of low sensitivity for detecting inferior wall lesions. While, this drawback could be overcome by using correction techniques during image reconstruction, this solution has adverse effects on specificity. They concluded that not only 360° data sampling did not show any advantage over 180° collection but also patients were more comfortable in the 180° mode by keeping only their left arm, not both, over their heads. Based on these findings, they recommended that application of 180° mode is advisable for SPECT study of the myocardium. Tamaki et al. (24) compared the spatial resolution, lesion contrast, and photon attenuation in cardiac scans with 180° orbit and those with 360° orbit. While photon attenuation was greater in the line source located deep in the water for 180° mode SPECT, this was not a significant finding in the clinical context. Perfusion defects were more detectable in 180° data collection and lesion contrast enhancement was higher. Based on the results of their study, they concluded that 180° mode offers a more effective method of imaging to assess patients with CAD.

During myocardial perfusion imaging, patients with CAD are required to perform a sufficient amount of exercise to reach maximum predicted heart rate in order to overcome the low diagnostic sensitivity of the scan (25,26). In case of insufficient exercise test, combining a dipyridamole stimulus is one of the options to provide sufficient diagnostic sensitivity (27). In this study, EDV, ESV and EF values were assessed after stress induction by either exercise or dipyridamole to compare the effects of these two types of stress on myocardium studies. The data revealed that there were no significant differences between the group with stress induced by exercise and the group with stress imposed by dipyridamole in terms of EDV, ESV and EF values. This finding was detected in both 180° and 360° data collection. Based on this finding, we may conclude that dipyridamole stress test could be applied as a suitable alternative in patients who are not able to perform exercise to increase their heart rate.

What’s more, the current study depicted that the R-R bin quantification between two types of data acquisitions did not differ significantly, which indicates the unchanged diastolic parameters in these two subgroups.

### Study Limitations

The main limitation of the current study was the low number of patients; therefore, it is recommended that a similar study with a larger population should be performed to compare these two scanning modes more comprehensively.

## CONCLUSION

180° data acquisition is suggested due to the patient comfort, shorter acquisition time and better image quality in comparison to 360° scanning mode, as reported by previous studies.

## Figures and Tables

**Table 1:**
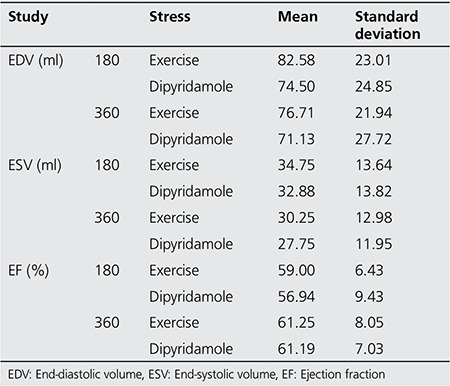
End-diastolic volume, end-systolic volume and ejection fraction measurements from 180° and 360° myocardial image perfusion in stress conditions

**Table 2:**
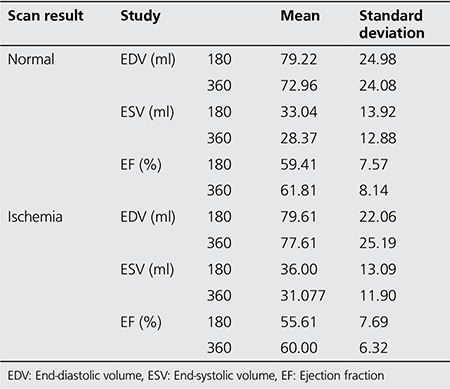
End-diastolic volume, end-systolic volume and ejection fraction from 180° and 360° myocardial perfusion imaging at normal and ischemia conditions
